# Acute-Onset Type 1 Diabetes in a Patient With Autoimmune Polyendocrine Syndrome Type 3A Following Influenza A Infection: A Case Report

**DOI:** 10.7759/cureus.104545

**Published:** 2026-03-02

**Authors:** Shota Doi, Tsuneaki Kenzaka, Nanami Kiriishi, Ryo Fujiwara, Hogara Nishisaki

**Affiliations:** 1 Department of Internal Medicine, Hyogo Prefectural Tamba Medical Center, Tamba, JPN; 2 Division of Community Medicine and Career Development, Kobe University Graduate School of Medicine, Kobe, JPN

**Keywords:** acute-onset type 1 diabetes mellitus, autoimmune polyendocrine syndrome type 3a, case report, hashimoto’s thyroiditis, hla-dr, hla-dr8, influenza a, type 1 diabetes

## Abstract

Type 1 diabetes is characterized by autoimmune destruction of pancreatic β-cells, driven by a combination of genetic susceptibility and environmental triggers. Influenza A virus infection has been reported to increase the risk of developing type 1 diabetes.

A 63-year-old Japanese woman with underlying Hashimoto’s disease contracted influenza one month prior. After contracting influenza A, she experienced persistent dry mouth and fatigue and presented to our outpatient clinic. She was diagnosed with diabetic ketoacidosis (DKA) caused by acute-onset type 1 diabetes. Given the coexistence of Hashimoto’s thyroiditis, she was diagnosed with autoimmune polyendocrine syndrome (APS) type 3A. DKA resolved with intravenous insulin and fluid therapy, and the patient was discharged on a basal-bolus insulin regimen. Clinicians should consider the possibility of type 1 diabetes in patients with autoimmune thyroid disease who develop hyperglycemic symptoms, such as polydipsia, polyuria, or weight loss, following influenza infection.

## Introduction

Type 1 diabetes is an autoimmune disorder in which pancreatic β-cells are progressively destroyed, ultimately resulting in absolute insulin deficiency [1]. Individuals with a genetic predisposition may develop autoimmune responses against pancreatic β-cells when exposed to environmental triggers, resulting in progressive β-cell loss [2]. Once the remaining β-cell mass falls below a critical threshold, hyperglycemia becomes clinically apparent [2]. Viral infections or flu-like symptoms are frequently observed prior to the onset of type 1 diabetes, suggesting that cytokine- and chemokine-mediated inflammatory responses may contribute to disease development [2].

A recent cohort study demonstrated that the incidence of new-onset type 1 diabetes within 180 days after influenza infection is 1.30-fold higher than during other periods [3]. Type 1 diabetes also frequently coexists with other autoimmune diseases, including thyroid disorders, and is thought to be influenced by shared genetic backgrounds and inflammatory environments [4]. Genetic susceptibility plays a crucial role in the development of type 1 diabetes and autoimmune polyendocrine syndromes (APS) [5]. In particular, human leukocyte antigen (HLA) class II genes are strongly associated with autoimmune diabetes and the clustering of autoimmune endocrine diseases. Such shared genetic backgrounds are thought to predispose individuals to the coexistence of autoimmune thyroid disease and type 1 diabetes [6].

APS is a group of disorders characterized by the coexistence of multiple autoimmune endocrine diseases and is classified into four major types (APS-1 to APS-4) based on organ involvement [6]. APS type 3 is defined by autoimmune thyroid disease without adrenal insufficiency and is further subclassified, with type 3A indicating coexistence with type 1 diabetes mellitus.

Although type 2 diabetes is more common in adults, it is primarily characterized by insulin resistance rather than autoimmune β-cell destruction. In contrast, type 1 diabetes results from autoimmune-mediated loss of pancreatic β-cells, a distinction that is essential when considering APS such as APS type 3A.

Autoimmune diseases often share common immunological mechanisms, and involvement of multiple endocrine organs is not uncommon. In this context, autoimmune thyroid disease and type 1 diabetes may coexist as part of APS, while environmental factors such as viral infections have been proposed to precipitate disease onset in genetically susceptible individuals.

In this report, we present a case of acute-onset type 1 diabetes that developed shortly after influenza A infection in a patient with underlying Hashimoto’s thyroiditis and impaired glucose tolerance.

## Case presentation

A 63-year-old Japanese woman presented with dry mouth and fatigue. Three years prior, she had been diagnosed with Hashimoto's thyroiditis with normal thyroid function and had been attending regular check-ups at our hospital every six months. Hashimoto's disease was diagnosed based on positive anti-thyroid peroxidase antibody and diffuse thyroid enlargement. At the time of diagnosis of Hashimoto’s thyroiditis, three years earlier, the patient was also found to have impaired glucose tolerance and was followed regularly. Prior to influenza infection, casual plasma glucose levels during outpatient visits were approximately 120-130 mg/dL, and glycated hemoglobin levels had remained stable between 5.8% and 6.0%. Type 1 diabetes-related autoantibodies had not been assessed before influenza onset. Body weight had remained essentially unchanged during the three years preceding influenza infection, and serum amylase levels had consistently been within the normal range (60-80 U/L). Three months prior to her visit, her HbA1c was 6.0%.

Thirty days before presentation, the patient developed a fever and was diagnosed with influenza A infection, for which she received oseltamivir for five days. The fever resolved within two days; however, fatigue persisted. Approximately 10 days after influenza onset, fatigue further worsened and was accompanied by thirst. These symptoms persisted, and polyuria and polydipsia subsequently developed. In addition, the patient experienced a weight loss of 5 kg over the course of one month. Because of persistent thirst and fatigue, she presented to the outpatient clinic.

Her past medical history included Hashimoto's thyroiditis, impaired glucose tolerance, dyslipidemia, osteoporosis, and cervical cancer (total hysterectomy at age 45). Her current medications are alendronate 100 mg/month and eldecalcitol 75 µg/day. There is no family history of diabetes or endocrine disorders.

Vital signs on presentation were as follows: Glasgow Coma Scale E4V5M6, temperature 36.2°C, peripheral oxygen saturation 99% on room air, blood pressure 138/78 mmHg, respiratory rate 16 breaths/min without Kussmaul respiration, and pulse rate 82 beats/min (regular). She measured 155 cm in height and weighed 50 kg, corresponding to a body mass index of 20.8 kg/m².

Examination findings included normal conjunctival coloration without pallor or icterus. The oral mucosa was dry. The thyroid gland showed no enlargement or tenderness. Cardiopulmonary examination was normal. The abdomen was soft and flat, with no associated pain or tenderness. There was no evidence of peripheral edema. Capillary refill time was two seconds. The skin appeared dry, without hyperpigmentation or rash.

Laboratory findings upon admission are summarized in Table 1. The results included white blood cells 4300/μL, hemoglobin 12.8 g/dL, C‑reactive protein 0.02 mg/dL, plasma glucose 437 mg/dL, HbA1c 10.9%, total ketone bodies 7770 µmol/L, acetoacetic acid 1399 µmol/L, and 3-hydroxybutyric acid 6371 µmol/L. Thyroid function tests showed thyroid-stimulating hormone 1.13 µIU/mL and free thyroxine 1.21 ng/dL. 

**Table 1:** Laboratory data upon admission

Parameter	Recorded value	Standard value
Complete Blood Count		
White blood cell count	4300 /µL	4500-7500 /µL
Hemoglobin	12.8 g/dL	11.3-15.2 g/dL
Platelet count	25.0 × 10^4^/µL	13-35 × 10^4^/µL
Biochemistry		
C-reactive protein	0.02 mg/L	≤0.60 mg/dL
Total protein	7.3 g/dL	6.9-8.4 g/dL
Albumin	4.5 g/dL	3.9-5.1 g/dL
Total bilirubin	1.0 mg/dL	0.2-1.2 mg/dL
Aspartate aminotransferase	24 U/L	11-30 U/L
Alanine aminotransferase	38 U/L	4-30 U/L
Lactase dehydrogenase	215 U/L	109-216 U/L
Amylase	71 U/L	44-132 U/L
Creatine kinase	97 U/L	40-150 U/L
Blood urea nitrogen	9.7 mg/dL	8-20 mg/dL
Creatinine	0.50 mg/dL	0.63-1.03 mg/dL
Sodium	137 mEq/L	136-148 mEq/L
Potassium	3.9 mEq/L	3.6-5.0 mEq/L
Chloride	101 mEq/L	98-108 mEq/L
Glucose	437 mg/dL	70-109 mg/dL
Hemoglobin A1c	10.9%	5.6-5.9%
Acetoacetic acid	1399 µmmol/L	28-46 µmmol/L
3-hydroxybutyric acid	6371 µmmol/L	0-74 µmmol/L
Thyroid-stimulating hormone	1.13 µIU/mL	0.61-4.23 µIU/mL
Free thyroxine (FT4)	1.21 ng/dL	0.7-1.48 ng/dL
Anti‑glutamic acid decarboxylase autoantibody (GAD)	≤5.0 U/mL	≤5.0 U/mL
Anti‑Insulinoma-associated protein-2 autoantibody (IA-2)	≤0.6 U/mL	≤0.6 U/mL
Insulin autoantibody (IAA)	0.8 U/mL	0-0.3 U/mL
Islet cell antibody (ICA)	+	-
Zinc transporter 8 antibody (ZnT8)	≤15.0 U/mL	≤15.0 U/mL
Arterial Blood Gas (Room Air)		
Potential hydrogen (pH)	7.261	7.35-7.45
Partial pressure of oxygen	73.0 mmHg	80.0-100 mmHg
Partial pressure of carbon dioxide	28.8 mmHg	35.0-45.0 mmHg
Bicarbonate ion (HCO_3_^-^)	12.9 mmol/L	20.0-26.0 mmol/L
Lactic acid	1.1 mmol/L	0.5-2.0 mmol/L
Anion gap	16.9	8-14
Urinalysis		
Specific gravity	1.015	1.005-1.030
pH	5.5	4.5-8.0
Ketone bodies	3+	

Arterial blood gas analysis on room air demonstrated pH 7.261, arterial oxygen partial pressure (PaO₂) 73.0 mmHg, arterial carbon dioxide partial pressure (PaCO₂) 28.8 mmHg, plasma bicarbonate concentration (HCO₃⁻) 12.9 mmol/L, lactate 1.1 mmol/L, and an anion gap of 16.9. Urine testing revealed a specific gravity of 1.015, with a pH of 5.5 and strong ketonuria (3+).

Following her influenza infection, her HbA1c had increased dramatically from 6% to 10.9% within this brief interval. Based on laboratory findings showing an HbA1c of 10.9%, a random blood glucose level of 437 mg/dL, positive urinary and serum ketone bodies, and metabolic acidosis with a pH of 7.261, she was diagnosed with diabetic ketoacidosis (DKA).

Abdominal and chest computed tomography showed no findings of pancreatitis, and no malignant tumors were detected in the pancreas or other organs.

Fluid resuscitation with potassium-containing extracellular solution was initiated, followed by continuous intravenous insulin infusion at 0.1 U/kg/h. By hospital day 2, the DKA had resolved rapidly, allowing discontinuation of the insulin infusion.

Once insulin infusion therapy was terminated, the patient was transitioned to subcutaneous insulin, receiving insulin aspart (4 units after each meal) in combination with insulin glargine (6 units at bedtime). Insulin doses were subsequently modified in response to blood glucose levels. By hospital day 10, testing indicated a profound reduction in endogenous insulin production, with a fasting C‑peptide concentration of 0.13 ng/mL (reference range: 0.62-2.54 ng/mL), a C‑peptide index of 0.1, and daily urinary C‑peptide excretion of 7 µg (reference range: 45-117 µg/day).

In the blood sample obtained at admission, testing for islet-related autoantibodies revealed positivity for insulin autoantibodies (IAAs) and islet cell antibodies (ICAs). Based on these findings, the patient met all diagnostic criteria for acute-onset type 1 diabetes mellitus (autoimmune) [7]: (1) development of diabetic ketosis or ketoacidosis within approximately three months of hyperglycemic symptoms (thirst, polydipsia, polyuria, and weight loss); (2) requirement for continuous insulin therapy after diagnosis; and (3) positivity for anti-islet autoantibodies.

Furthermore, morning ACTH and cortisol levels were within normal ranges, and no clinical features of adrenal insufficiency were observed. Therefore, in addition to her known Hashimoto’s thyroiditis, the patient was considered to have newly developed acute-onset type 1 diabetes, fulfilling the diagnostic criteria for APS type 3A (Table 2) [6].

**Table 2:** Classification of autoimmune polyendocrine syndromes (APS) modified from Neufeld Source: [6]

Type	Major component diseases
APS-1 or APECED (autoimmune polyendocrine-candidiasis-ectodermal-dystrophy)	Chronic mucocutaneous candidiasis, chronic hypoparathyroidism, and Addison’s disease (at least two out of three)
APS-2 or Schmidt’s syndrome	Addison’s disease + thyroid autoimmune disease and/or diabetes mellitus type 1
APS-3 (excluding Addison’s disease)	Thyroid autoimmune disease + diabetes mellitus type 1: Type 3A
	Chronic atrophic gastritis or pernicious anemia: Type 3B
	Vitiligo, alopecia, or myasthenia gravis: Type 3C
APS-4	Any other possible association of autoimmune diseases

At discharge, the patient was receiving insulin aspart at 12 units following breakfast and 10 units after both lunch and dinner, in combination with 10 units of insulin glargine at night. Blood glucose levels subsequently stabilized, allowing discharge on the 14th hospital day in an ambulatory state.

Because of the rapid deterioration in glycemic control, an evaluation for malignancy was performed. A malignancy workup - including ultrasonography, computed tomography, upper gastrointestinal endoscopy, and colonoscopy - showed no abnormalities. In addition, upper gastrointestinal endoscopy showed no evidence of autoimmune gastritis, which is frequently associated with APS.

Given the abrupt onset of insulin deficiency and the coexistence of autoimmune thyroid disease, HLA typing was performed to support the diagnosis of autoimmune type 1 diabetes and APS. HLA typing demonstrated that the patient carried HLA-DR8 and HLA-DR9.

She continued outpatient treatment with once-daily basal insulin and thrice-daily rapid-acting insulin, but her HbA1c remained in the 8% range over the six-month period. After discharge, her insulin requirements remained stable, with insulin glargine at 7-8 units per day and a total daily insulin aspart dose of 20-25 units. Three months after discharge, her fasting serum C‑peptide level remained low at 0.22 ng/mL (reference range: 0.62-2.54 ng/mL), indicating persistent severe insulin secretory failure.

Thyroid function has remained normal. Anti‑adrenal antibodies were confirmed to be negative, and no abnormalities in adrenocorticotropic hormone or cortisol levels suggestive of adrenal insufficiency have been observed. However, if the development of Addison’s disease is identified in the future, the diagnosis may need to be revised to APS type 2; therefore, careful long‑term follow-up is planned.

## Discussion

This case involved acute-onset type 1 diabetes following influenza A infection, characterized by complete depletion of insulin secretion. In addition, the patient had pre-existing Hashimoto’s thyroiditis, leading to a diagnosis of APS type 3A. Although previous reports have described insulin secretory failure and the onset of type 1 diabetes after influenza A infection, to our knowledge, this is the first documented case in which APS was diagnosed concurrently. Differential diagnoses included fulminant type 1 diabetes and latent autoimmune diabetes in adults (LADA). However, the marked elevation in HbA1c, positivity for islet-related autoantibodies, progressive symptom development over several weeks, and persistently reduced C‑peptide levels argue against fulminant type 1 diabetes, while the abrupt onset, with DKA and immediate insulin dependence, makes LADA unlikely.

Type 1 diabetes is a form of diabetes caused by insulin deficiency resulting from destructive lesions of pancreatic β-cells, and both genetic and environmental factors contribute to its pathogenesis [1,2]. Among these, genetic factors, such as HLA, are considered particularly important.

HLA is regarded as the strongest genetic predisposing factor for type 1 diabetes. In the Japanese population, HLA-DR4 and DR9 are known to confer susceptibility, whereas DR2 confers resistance [8]. Recent studies have also demonstrated that the DR8 haplotype, which is rare in the general population, is associated with high disease susceptibility [9]. In the present case, outpatient testing revealed the presence of HLA-DR8 and DR9, suggesting a high genetic susceptibility to type 1 diabetes.

A variety of environmental factors are known to contribute to the development of type 1 diabetes, and recent studies have demonstrated that influenza A virus can induce pancreatitis and diabetes in animal models [10]. Among interferon-gamma (IFN-γ)-inducible chemokines, C-X-C motif chemokine ligand (CXCL)9 and CXCL10 show the greatest increases in pancreatic islet cells following influenza A infection [10]. CXCL10 is thought to contribute to the onset of autoimmune islet destruction by attracting monocytes, T lymphocytes, and natural killer (NK) cells, and by promoting the migration of antigen-specific lymphocytes [10]. Furthermore, the presence of islet-associated autoantibodies (IAAs) several years before the clinical onset of type 1 diabetes is not uncommon [11], and it has been suggested that influenza infection may trigger pancreatitis, subsequently leading to the development of type 1 diabetes over the course of several months. In this case as well, based on previous research findings, it was presumed that the patient - who had a genetic predisposition to type 1 diabetes due to HLA susceptibility - developed the disease approximately one month before presentation, following influenza infection that altered the inflammatory milieu through chemokine activation.

Hashimoto’s disease represents an autoimmune thyroid disorder that commonly occurs in association with other autoimmune diseases [12]. Epidemiological studies have shown that patients with Hashimoto’s disease have a substantially increased prevalence of comorbid autoimmune conditions, including type 1 diabetes mellitus, vitiligo, rheumatoid arthritis, and Sjögren’s syndrome, compared with the general population [12]. The simultaneous presence of multiple autoimmune disorders is termed APS, which is categorized into four types based on the pattern of organ involvement [6]. In APS type 3, autoimmune thyroid disease is accompanied by other autoimmune conditions and is subclassified into types 3A through 3D according to the associated diseases [6]. Among these categories, type 3A - characterized by coexistence with type 1 diabetes - is the most common. In the present case, these clinical features led to the diagnosis of APS type 3A.

Epidemiological data indicate that autoimmune thyroid disease is present in several percent to the low‑teen range among Japanese patients with type 1 diabetes [5]. This observation suggests a contribution of HLA‑dependent genetic factors to APS susceptibility. In particular, DR9‑positive type 1 diabetes is often accompanied by autoantibodies, including anti-glutamic acid decarboxylase (anti‑GAD) and anti‑thyroid antibodies, pointing to a potential role of DR9 in promoting autoantibody production [12]. In contrast, IAAs are more frequently detected in DR4‑positive cases [12]. Furthermore, elevated CXCL10 expression in serum or tissues has been documented in multiple organ‑specific autoimmune conditions, such as autoimmune thyroiditis, type 1 diabetes, rheumatoid arthritis, and systemic lupus erythematosus [12]. These findings suggest that, beyond shared genetic predisposition, the immunological microenvironment - particularly chemokine signaling - may contribute to the clustering of autoimmune diseases in type 1 diabetes.

It should be emphasized that the present case demonstrates a temporal association rather than a definitive causal relationship between influenza A infection and the onset of type 1 diabetes. Previous epidemiological studies and experimental models have suggested an association between viral infections and autoimmune diabetes, although similar case reports describing concurrent APS remain limited.

This case also highlights a potential diagnostic challenge. In adult patients, particularly those with impaired glucose tolerance, acute‑onset type 1 diabetes may be misclassified as type 2 diabetes, especially by less experienced clinicians or in settings with limited access to immunological testing. Awareness of rapid insulin deficiency, ketosis, and coexisting autoimmune disease is therefore essential to avoid delayed or inappropriate diagnosis.

## Conclusions

We describe a rare case of acute‑onset type 1 diabetes that developed shortly after influenza A infection in a patient with pre‑existing Hashimoto’s thyroiditis. The concurrent diagnosis of APS type 3A in this context is highly unusual and underscores the potential for viral infections to accelerate autoimmune processes in genetically susceptible individuals.

Clinicians should consider type 1 diabetes when patients with autoimmune thyroid disease develop new hyperglycemic symptoms following influenza infection. Early recognition of manifestations such as polydipsia, polyuria, weight loss, or persistent fatigue is essential to prevent severe metabolic decompensation. This case highlights the importance of careful monitoring in individuals with autoimmune backgrounds, particularly when environmental triggers such as viral infections are present.

## References

[1] Filippi CM, von Herrath MG (2008). Viral trigger for type 1 diabetes: pros and cons. Diabetes.

[2] Eisenbarth GS (1986). Type I diabetes mellitus. A chronic autoimmune disease. N Engl J Med.

[3] Nishioka Y, Noda T, Okada S (2021). Association between influenza and the incidence rate of new-onset type 1 diabetes in Japan. J Diabetes Investig.

[4] Warncke K, Fröhlich-Reiterer EE, Thon A, Hofer SE, Wiemann D, Holl RW (2010). Polyendocrinopathy in children, adolescents, and young adults with type 1 diabetes: a multicenter analysis of 28,671 patients from the German/Austrian DPV-Wiss database. Diabetes Care.

[5] Frommer L, Kahaly GJ (2021). Type 1 diabetes and autoimmune thyroid disease - the genetic link. Front Endocrinol (Lausanne).

[6] Husebye ES, Anderson MS, Kämpe O (2018). Autoimmune polyendocrine syndromes. N Engl J Med.

[7] Kawasaki E, Maruyama T, Imagawa A (2014). Diagnostic criteria for acute-onset type 1 diabetes mellitus (2012): report of the Committee of Japan Diabetes Society on the research of fulminant and acute-onset type 1 diabetes mellitus. J Diabetes Investig.

[8] Kawabata Y, Ikegami H, Awata T (2009). Differential association of HLA with three subtypes of type 1 diabetes: fulminant, slowly progressive and acute-onset. Diabetologia.

[9] Ina Y, Kawabata Y, Sakamoto R, Sekiguchi N, Ikegami H (2017). Rare human leukocyte antigen genotype in two siblings with type 1 diabetes in a Japanese family clustered with type 1 diabetes. J Diabetes Investig.

[10] Capua I, Mercalli A, Pizzuto MS (2013). Influenza A viruses grow in human pancreatic cells and cause pancreatitis and diabetes in an animal model. J Virol.

[11] Hummel M, Bonifacio E, Schmid S, Walter M, Knopff A, Ziegler AG (2004). Brief communication: early appearance of islet autoantibodies predicts childhood type 1 diabetes in offspring of diabetic parents. Ann Intern Med.

[12] Fallahi P, Ferrari SM, Ruffilli I (2016). The association of other autoimmune diseases in patients with autoimmune thyroiditis: review of the literature and report of a large series of patients. Autoimmun Rev.

